# Health Workforce Equity in Urban Community Health Service of China

**DOI:** 10.1371/journal.pone.0115988

**Published:** 2014-12-31

**Authors:** Rui Chen, Yali Zhao, Juan Du, Tao Wu, Yafang Huang, Aimin Guo

**Affiliations:** 1 School of General Practice and Continuing Education, Capital Medical University, Beijing, 100069, P.R. China; 2 Beijing An Zhen Hospital, Capital Medical University, Beijing, P. R. China; Örebro University, Sweden

## Abstract

**Objectives:**

To reveal the equity of health workforce distribution in urban community health service (CHS), and to provide evidence for further development of community health service in China.

**Methods:**

A community-based, cross-sectional study was conducted in China from September to December 2011. In the study, 190 CHS centers were selected from 10 provinces of China via stratified multistage cluster sampling. Human resources profiles and basic characteristics of each CHS centers were collected. Lorenz curves and Gini Coefficient were used to measure the inequality in the distribution of health workforce in community health service centers by population size and geographical area. Wilcoxon rank test for paired samples was used to analyze the differences in equity between different health indicators.

**Results:**

On average, there were 7.37 health workers, including 3.25 doctors and 2.32 nurses per 10,000 population ratio. Significant differences were found in all indicators across the samples, while Beijing, Shandong and Zhejiang ranked the highest among these provinces. The Gini coefficients for health workers, doctors and nurses per 10,000 population ratio were 0.39, 0.44, and 0.48, respectively. The equity of doctors per 10,000 population ratio (G = 0.39) was better than that of doctors per square kilometer (G = 0.44) (P = 0.005). Among the total 6,573 health workers, 1,755(26.7%) had undergraduate degree or above, 2,722(41.4%)had junior college degree and 215(3.3%) had high school education. Significant inequity was found in the distribution of workers with undergraduate degree or above (G = 0.52), which was worse than that of health works per 10000 population (P<0.001).

**Conclusions:**

Health workforce inequity was found in this study, especially in quality and geographic distribution. These findings suggest a need for more innovative policies to improve health equity in Chinese urban CHS centers.

## Introduction

Health equity is an explicit goal for health care reform around the world, numerous countries have pursued this goal to ensure universal and equitable access of healthcare [1], [2], [3], [4] . In the 58th World Health Assembly (2005), all member states were encouraged to achieve universal healthcare coverage, thereby achieving equity in access [5]. At present, financial, material, and human resources are three major inputs that national health reform have to deal with in China. Undoubtedly, money, drugs, and equipment are needed, but these inputs are meaningless without effective workforce. However, it is fairly difficult to allocate the limited human resource due to the dynamically changing nature of this resource [6]. In many countries, there are substantial evidences which revealed wide inequity of health workforce in primary health care [7], [8].

In China, community health service (CHS) organizations are designated as the first level of contact to the basic primary health care in urban areas [9]. These organizations serve a population of 408 million urban residents, accounting for about 30% of the Chinese population [10]. To achieve the goal of health care for all and to reduce the burden of health care expenses, the Chinese government launched the CHS program in 1997 [11]. At present, CHS organizations including CHS centers and their affiliated stations have become essential parts of the primary health care institutions in urban areas. These organizations specify their service scope and goals based on different circumstance of communities. Since China's new medical reform positioned community health services as one of the top five priorities in 2009, vigorous efforts have been put into building a community-based health system [12]. By the end of 2011, a total of 32,860 CHS centers and stations had been set up in China, and the CHS network was preliminarily established [10]. General practitioner is the core of primary health care system, the rationality of its workforce distribution is the key point to ensure the equity of CHS [13]. On July 7, 2011, the State Council put forward a target of 2∼3 general practitioners per ten thousand people by the year 2020 [12].

With increasing government input, the infrastructure has been greatly improved in CHS. However, there is still a shortage and inequitable distribution of health workers, which becomes the bottleneck for the sustainable development of CHS [9]. The realization of the health service equity depends significantly on the reasonable distribution of human resources in primary health services. In many countries, health workers tend to serve in economically developed cities rather than poverty-stricken areas, therefore there is a severe inequity in the distribution of health workforces [14]. What's more, medical students with higher degree prefer to go to specialized hospitals, rather than communities [15]. Therefore how to allocate the limited human resources properly to promote equity is a fundamental goal of healthcare reform worldwide, especially in developing countries such as China.

Recently, although several studies examined the extent of inequity, they mainly focused on specific health outcomes, health care utilization, and health economic input [16], [17]. Though there were many studies [18], [19] on equity in health workforces, most of these studies focused on urban-rural inequity not inter-urban inequity. In fact, the construction of primary health care in rural areas of China is based on the CHS development experience. In other words, we can improve the equity of human resource in the rural primary health care by taking advantage of the experience of urban CHS. However, there is little evidence regarding the distribution of health workforces in CHS centers which provide basic health care for people at primary levels. Meanwhile, the majority of the studies focused on equity in quantity rather than in quality [20]. In this study, we analyzed China's current health workforce in terms of quantity and quality distribution in urban CHS centers. This study attempt to give advices and references of the optimal allocation of human resources to promote the development of CHS for the Chinese government.

## Methods

### Sampling methods

There are 31 provinces (including five autonomous regions and four municipalities) in Mainland China, which were divided into eastern, central and western regions according to economic development. The health administrative agencies made slight adjustment in the division based on the actual development of health service institutions, thus there are 10, 9 and 12 provinces (or autonomous regions, municipalities) in eastern, central and western region, respectively.

**Figure 1:**
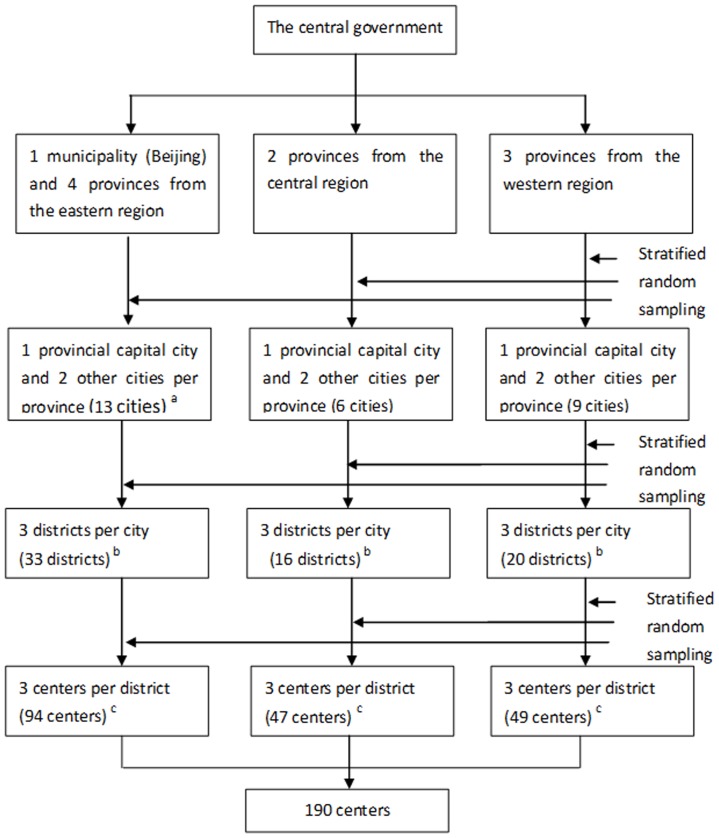
Sampling flow of community health services centers. a: Beijing is a municipality with no affiliated city. Dalian is selected in Liaoning province instead of its capital city, because it is one of the five cities (Xiamen, Ningbo, Qingdao, Shenzhen, and Dalian) specially designated in the state plan which is similar in size and economic situation to a provincial capital. b: It is plan to sample 3 districts per city in the design, while some cities selected had less than 3 districts. c: It is plan to sample 3 centers per district in the design, while some districts selected had less than 3 centers.

A community-based, cross-sectional study was conducted from September to December in 2011 by the National Health and Family Planning Commission, China. Ten provinces including 28 cities, 69 districts and 192 CHS centers were selected by stratified multi-stage cluster sampling based on location, economic characteristics, and the level of development of CHS. The sampling process is shown in Fig. 1. First, ten provinces were extracted from the 31 provinces by three regions. These provinces were Beijing, Zhejiang, Liaoning, Shandong and Fujian (eastern region), Hebei and Hunan (central region), Guizhou, Guangxi and Ningxia province (western region). Second, we further selected three cities from each province. The provincial capital cities must be selected. The other two cities of each province were randomly selected by stratification according to high and low GDP clusters (below median and above median) [21]. Third, all urban districts of each selected city were divided into 3 strata according to GDP in 2012, and the districts were randomly selected by strata. Finally, three CHS centers were randomly selected from each selected district. In summary, 10 provinces, 20 cities, 69 districts and 192 CHCs were selected for the survey

### Data collection

Data were collected from September 2011 to December 2011 by data collection questionnaire that completed by directors of selected CHS centers, with supervision of researchers. Following data were included in the data collection form: geographic area of district jurisdiction, residential population of district, number of health workers of the CHS centers and its affiliated stations as well as detailed human resources profiles. Information about the number of practicing doctors and registered nurses at province level were drawn from China Health Statistics Digest (2012) published by the State Statistics Bureau [10].

A uniform questionnaire had been used in this investigation, and was completed by directors of each CHS. Administrative officers and directors of CHS organizations were recruited from each city to conduct this survey along with the researchers. The survey process was standardized by training among all investigators before data collection. One hundred and ninety-two questionnaires were issued and recovered. Of these, two questionnaires were discarded because of missing data or logical error, with an overall response rate of 98.96%. Thus, data of 190 CHS centers was collected in our study.

### Ethics Statement

Ethical approval was obtained from the Medical Ethics Committee of Capital Medical University, Beijing, China. We have not access the health works except the directors of each CHS center, and no sources of potential harm to the participants were apparent. T data were gathered base on the whole CHS centers. The details data such as age, gender, and medical educational background of each health workers were not collected in our study.

### Assessment of Inequity

We used Lorenz curves and Gini Coefficient to measure the inequalities in the distribution of health workforce in CHS centers by population size and geographical area. Both the Lorenz curves and Gini Coefficient have been widely used to assess the distributional properties of income and wealth, and have been applied medical and health services [19].

The Lorenz curve is a graphical display of the distribution of the cumulative percent of events by the cumulative percent of people in the population [22]. We draw a set of axes in which the cumulative proportion of the population or area measured along the x-axis while the cumulative percentage of health variable displayed along the y-axis. The individuals on the x-axis are ranked according to the variable on the y-axis. A straight line rising at an angle of 45° from the origin on the graph will indicate perfect equality. The greater the distance from the perfect equality line, the greater the inequality is [23].

The Gini Coefficient is a complementary way of presenting information about inequality [24]. It is the ratio of the area between the Lorenz Curve and the line of perfect equality (numerator) and the whole area under the line of absolute equality (denominator) [25]. If the Lorenz curve is represented by the function *Y = L(X)*, the area under the Lorenz curve (*B*) can be calculated with integration:
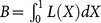
. Then the Gini index is *G = 1–2B*. In terms of this statistic, the greater the number, which ranges between 0.0 and 1.0, the higher the degree of inequality of health workforce. The currently accepted standard is followed by: Gini≤0.2 denotes absolute equality, 0.2<Gini means a relative equality, 0.3<Gini indicates an basically reasonable; 0.4<Gini means a bigger difference, while lager than 0.5 means great gap [26].

### Data Analysis

The total health workers per 10000 population ratio (HWPR), doctors per 10000 population ratio (DPR), nurses per 10000 population ratio (NPR), doctors per square kilometer (DSK), and ratio of doctors to nurses were calculated of each selected CHS. Descriptive statistics of personnel structure and educational background structure in the 190 CHS centers were performed using mean, median, and interquartile range.

We measured total inequalities in the distribution of total health workers, doctors, and nurses per 10000 population or per square kilometer within 190 CHS centers by Lorenz curve and Gini coefficient.

Subsequently, we measured inequalities in the distribution of total health workers, doctors, and nurses per 10000 population or per square kilometer within each province by Lorenz curve and Gini coefficient. Wilcoxon signed-rank test was used to test whether there is a difference between the Gini coefficients of different indicators.

Data were entered twice independently using Epidata3.0 and checked for errors to enhance the accuracy. Lorenz curves were plotted using Microsoft Excel 2007. Gini coefficients were calculated using MATELAB 7.1: a software system for numerical computations and graphics. SPSS 17.0 was used for statistical analyses. Two-tailed p values and 95% confidence intervals were reported for all statistical tests, with p<0.05 considered statistically significant.

## Results

In the 190 CHS centers, there were 6,573 health workers employed, including 2,900 doctors and 2,071 nurses who provided primary health care to 8.92 million people. On average, there were 7.37 health workers, including 3.25 doctors and 2.32 nurses per 10,000 population ratio.

Number of health workforce per 10,000 population ratio or per square kilometer varied greatly among CHS centers. Of the 190 CHS centers, the median of health workers per 10000 population ratio was 7.13 (range from 1.04 to 68.4). The maximum of doctors and nurses per 10,000 population ratio were 33.32 and 20.00 respectively, while minimum of them was zero (one CHS only has public health doctors who pursue the duty of maternal and children health care). Geographically, there was an average of 4.7 doctors per square kilometer in the 190 CHS centers, with the maximum number of 47.06 and the minimum number of zero. The Median ratio of doctors to nurses was 1.25∶1, with the highest and lowest ratio being 9∶1 and 1∶2.67, respectively (Those CHS centers with no doctor or nurse were excluded from this calculation.).

### Total equity in quantity

Table 1 showed the distribution of health workers, doctors, and nurses in the ten provinces of China. Substantial differences could be found among all indicators across the country. Beijing, Shandong and Zhejiang ranked the highest among these provinces.

**Table 1:** Health workforce per 10000 population ratio or per square kilometer in each province (n = 10).

Province	CHS (N)	Median(P_25_, P_75_)			
		HWPR	DPR	NPR	DSK
Beijing	7	11.16(4.90,13.33)	5.00(2.21,6.69)	4.04(1.53,6.00)	5.72(1.23,10.23)
Zhejiang	18	9.01(6.35,14.75)	4.76(3.06,8.84)	3.00(1.15,5.47)	2.77(0.65,8.03)
Hebei	26	7.60(5.37,14.71)	4.05(2.33,8.01)	2.64(1.52,4.67)	1.51(0.78,12.03)
Shandong	18	10.68(9.02,17.15)	3.85(2.94,7.36)	1.81(0.76,4.63)	3.58(1.58,5.61)
Liaoning	27	6.40(3.94,11.54)	3.20(2.12,4.76)	1.43(0.57,2.86)	2.00(0.56,4.17)
Guangxi	18	7.89(5.86,18.61)	3.00(2.24,9.18)	2.27(1.15,4.09)	2.87(1.21,6.17)
Fujian	24	6.95(4.83,10.52)	2.60(1.38,5.05)	0.56(0.20,1.82)	0.96(0.30,6.45)
Guizhou	21	6.52(3.91,19.54)	2.50(1.39,9.34)	0.64(0.00,5.06)	1.09(0.82,4.14)
Ningxia	10	6.47(4.75,9.84)	2.07(1.57,4.13)	1.25(0.74,1.93)	1.25(0.50,4.12)
Hunan	21	3.53(2.18,5.36)	0.98(0.58,1.96)	0.37(0.23,0.99)	0.52(0.15,2.78)

HWPR: health workers per 10,000 population ratio, DPR: doctors per 10,000 population ratio, NPR: nurses per 10,000 population ratio, DSK: doctor per square kilometer.

**Figure 2:**
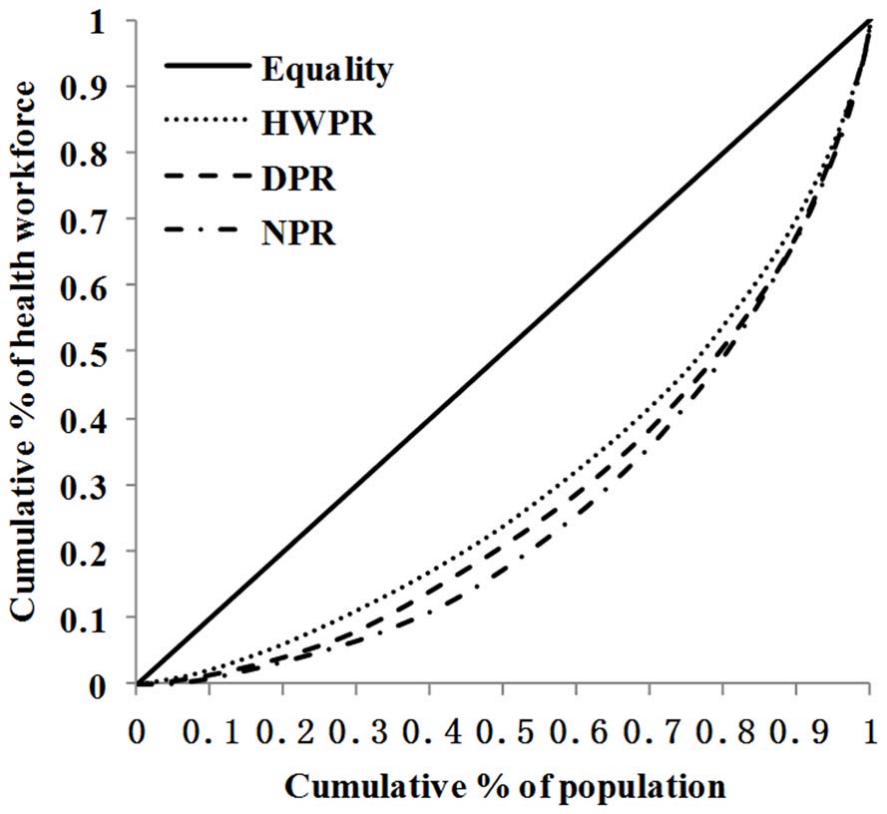
Lorenz curve of the distribution of health workforce per 10,000 population ratio in Chinese CHS. HWPR: health works per 10,000 population ratio, DPR: doctors per 10,000 population ratio, NPR: nurses per 10,00 population ratio. All CHS centers (n = 190) are represented in each curve.

The Lorenz curve concerning the distribution of health workers, doctors and nurses per 10,000 population ratio in the 190 CHS centers were demonstrated in Fig. 2, which showed that inequity exists in health workforce in CHS centers. The Gini coefficients for health workers, doctors and nurses per 10,000 population ratio were 0.39, 0.44, and 0.48, respectively. Wilcoxon matched-pairs ranks test of the 10 provinces showed that the inequality measured by nurses per 10,000 population ratio was greater than health works per 10000 population ratio (P = 0.007), while there was no significant difference between the Gini coefficients of health works per 10000 population ratio and doctors per 10,000 population ratio (P = 0.241).

**Figure 3:**
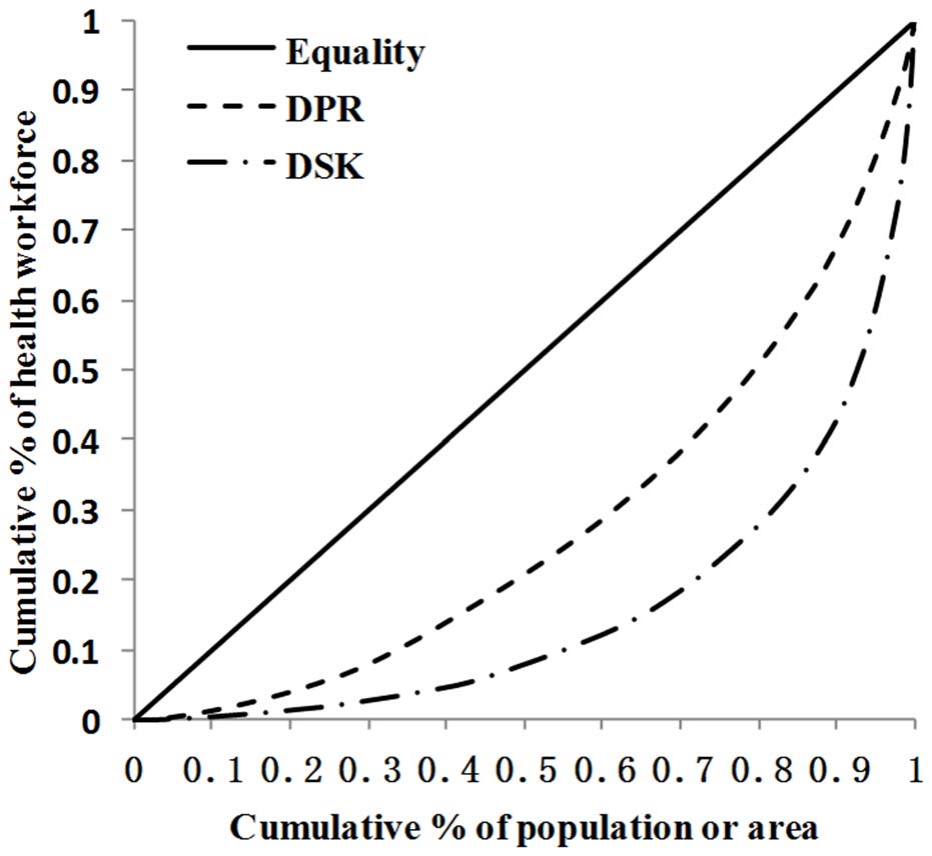
Lorenz curve of the distribution of doctors per 10000 population ratio and per square kilometer in Chinese CHS. DPR: doctors per 10000 population ratio, DSK: doctor per square kilometer. All CHS centers (n = 190) are represented in each curve.

Fig. 3 graphed the distribution of doctors per 10,000 population ratio and per square kilometer in the 190 CHS centers. The figure showed that the distribution of doctors per 10000 population ratio had greater Gini coefficient (0.68) than that of doctors per square kilometer (0.44). Wilcoxon matched-pairs ranks test showed that there was significant difference between the Gini coefficients of doctors per square kilometer and doctors per 10,000 population ratio (P = 0.005), which indicated that the inequality measured by doctors per square kilometer was greater than that of doctors per 10,000 population ratio.

### Total equity in quality

**Figure 4:**
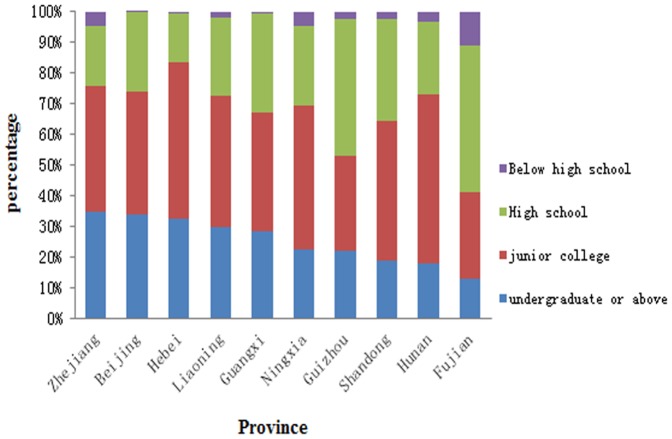
Distribution of total health workers by education level in each province (n = 10).

Among all the 6,573 health workers, 1,755(26.7%) had undergraduate degree or above, 2,722(41.4%)had associate degree, 215(3.3%)had only high school education, and 215(3.3%)had even lower educational level. The distribution of total health workers by education level in each province was show in Table 2 and Fig. 4.

**Table 2:** Distribution of total health workers by education level in each province (n = 10).

Province	Education level				Total
	undergraduate or above	junior college	High school	Below high school	
Beijing	210(34.1)	244(39.7)	160(26.0)	1(0.2)	615
Zhejiang	436(34.7)	515(41.0)	245(19.5)	59(4.7)	1255
Heibei	199(32.8)	309(50.9)	95(15.7)	4(0.7)	607
Shandong	103(19.1)	243(45.2)	180(33.5)	12(2.2)	538
Liaoning	244(29.8)	351(42.9)	207(25.3)	16(2.0)	818
Guangxi	186(28.6)	251(38.6)	211(32.4)	3(0.5)	651
Fujian	83(13.0)	180(28.1)	306(47.8)	71(11.1)	640
Guizhou	100(22.0)	140(30.8)	203(44.7)	11(2.4)	454
Ningxia	103(22.7)	211(46.6)	117(25.8)	22(4.9)	453
Hunan	91(18.1)	278(55.2)	119(23.6)	16(3.2)	504

**Figure 5:**
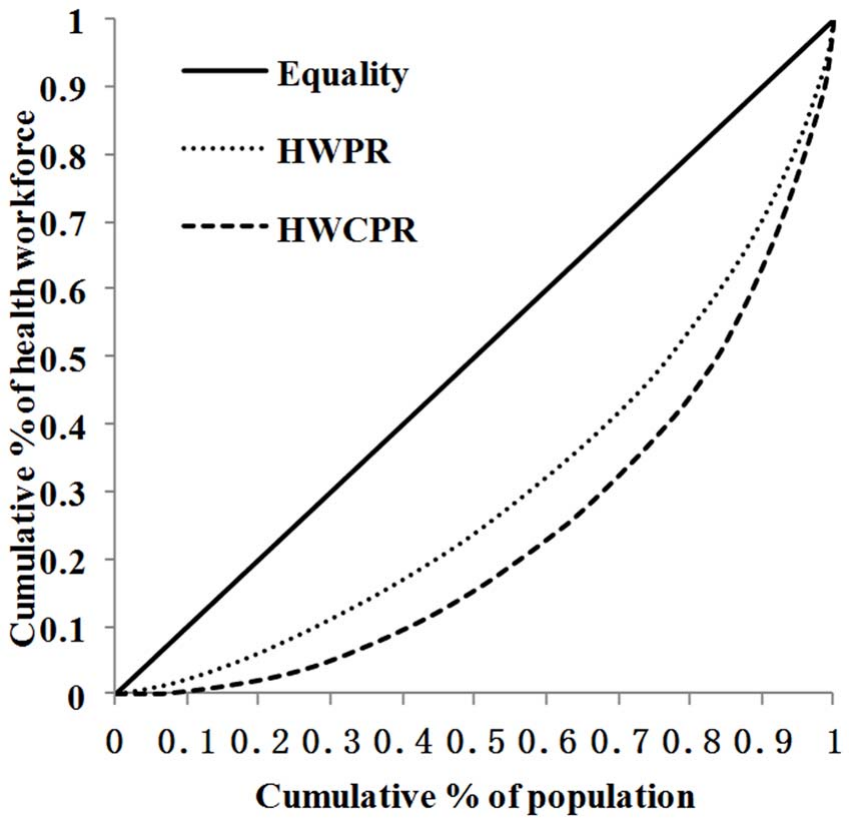
Lorenz curve of the distribution of total health workforce and health workers educated to college level or above per 10000 population ratio in Chinese CHS. HWPR: health works per 10000 population ratio; HWCPR: health workers educated to college level or above per 10000 population ratio.

Fig. 5 showed the distribution of total health workers per 10,000 population ratio and health workers with college level or above per 10,000 population ratio in the 190 CHS centers. The Gini coefficient for total health workers was 0.39, while that for health workers with college level or above was 0.52. Wilcoxon matched-pairs ranks test showed that there was significant difference between the Gini coefficients of health works per 10000 population ratio and health workers educated to college level or above per 10000 population ratio (P<0.001), which showed that the inequity measured by health workers educated to college level or above per 10000 population ratio is greater than that of health works per 10000 population ratio.

### Equity within provinces

We calculated the Gini coefficients of different indicators for each province (Table 3). It could be seen that the Gini coefficients of nurses per 10,00 population ratio and health works per 10000 population ratio were both greater than that of health works per 10000 population ratio within the 10 provinces (except Beijing), while the inequity of doctor per square kilometer was greater than doctors per 10,000 population ratio in each province.

**Table 3:** Gini coefficients of different indicators for each province (n = 10).

Province	HWPR	DPR	HWCPR	NPR	DSK
Beijing	0.341	0.335	0.328	0.404	0.477
Zhejiang	0.323	0.321	0.403	0.413	0.629
Heibei	0.439	0.470	0.491	0.498	0.691
Shandong	0.227	0.245	0.444	0.278	0.387
Liaoning	0.406	0.357	0.534	0.496	0.657
Guangxi	0.308	0.445	0.332	0.332	0.517
Fujian	0.321	0.393	0.536	0.443	0.667
Guizhou	0.507	0.549	0.746	0.527	0.655
Ningxia	0.258	0.216	0.269	0.247	0.575
Hunan	0.341	0.520	0.424	0.496	0.715

HWPR: health works per 10,000 population ratio, DPR: doctors per 10,000 population ratio, NPR: nurses per 10,000 population ratio, DSK: doctor per square kilometer.

## Discussion

China had undergone the most rapid industrialization and urbanization in the last few decades. As the foundation and entry point of China's urban health system, CHS had been established throughout the entire country [27]. The absolute number of health workers served in CHS centers has increased over the past 50 years [9]. However, quantity of CHS centers health workers is not sufficient to reflect the universal accessibility of health service, the distribution of human resources is also important. The fact that increasing supply of human resources does not necessarily means the decline of inequity has also been proved in other countries [28]. Since reform of urban and rural health systems in China are conducted separately, more attention has been paid to rural-urban differences and within rural equity [29], [30], [31]. Little has been done to estimate the health workforce distribution in urban CHS centers in China. This study assessed the equity in the distribution of health workforce in Chinese urban CHS centers in multiple provinces representing different economic levels and geographic regions.

### Inequity among eastern, central and western regions

Our research revealed that eastern provinces with the highest level of economic development had the highest health workload compared with other regions. For example, Beijing and Zhejiang in eastern region ranked top one and two on the quantity and quality of health workforce. The situation might mainly be explained by following two aspects. On the one hand, there is a strong relationship between the status of local economy and the development of CHS, the development of CHS centers in rich areas where government more financial investment and people pay more attention to primary health care, is generally faster than that of less developed areas. Beijing and Zhejiang are economically developed, and the governments give more financial support for the development of CHS centers [21]. And the higher income and better welfare will attract the more highly professional staffs to service. On the other hand, the introduction of general practice in developed areas is earlier compared with other areas, which makes the training of general practitioner and the development of CHS easier. The earliest university who introduced the concept of general practice and studied the development of it in China Mainland is the Capital Medical University. Besides, Sir Run Run Shaw Hospital, School of Medicine, Zhejiang University is the first class A tertiary hospital who set up the general medicine department in China Mainland.

However, the Chinese government has recognized the inequity between eastern and western areas and financing policies have been established to improve the situation [32]. Now special financial support has been given to central and western regions and mandatory reform tasks were assigned to these areas for the development of community health services and general practice training, while local governments in eastern regions are required to make plans and raise funds for the development of CHS in their regions. It can be seen that Hebei, as a province in the central region, has relatively faster development in CHS health workforces, which is partly benefited from the national financial support. On the contrary, the quality and quantity of health workers in Fujian province, where the economic development is unbalanced, is not ideal, despite its location in the affluent region of eastern China. Together, these findings indicated that the distribution of work forces was significantly influenced by the economic development levels and government subsidies. Targeted strategies made by the Chinese government for different economic base areas have certainly played a positive role in improving equity of health workforce in CHS centers.

### Equity in quantity

We found certain uneven distributions in health workers of Chinese CHS centers, especially among nurses and highly educated workers. The degrees of inequity in nurses were greater than that of doctors in our study. These findings corroborated results from an earlier study [33]. Because community health service was the first point of contact with the health system, the CHS centers should not be set to far away from residents. Equity at the geographic level is also significant for the utilization of community health service. Our study showed that there was greater inequality in the distribution of health workers per square kilometer than that of health workers per10000 population ratio. This inequity indicated that some remote residents might have to travel longer distances to receive primary health care than residents in densely populated areas [34]. These results suggested that the health service centers might set up more health stations for the less densely populated area, rather than a massive increase in the number of health workers. We also found Gini coefficient within-province varied from 0.3 to 0.5 (except Shandong and Ningxia), suggesting that inequality needs to be addressed at the provincial level. The most significant inequity was found in Guizhou, with a Gini greater than 0.5.

World Health statistics reported that there were 1.4 doctors and 2.8 nurses per 1000 people on global average in 2009. In China, number of doctors per 1000 population was 1.47 according to data in 2007, which exceeded the world average level. However, that there was still a serious shortage of nurses, with only 1.52 nurses per 1000 population. Our study showed that situation was even more unoptimistic in CHS, with a doctor to nurse ratio of 1.25∶1, which was consistent to the other reports [35]. It could be seen that more community nurses should be trained and recruited for the CHS centers to optimize the composition of the GP team.

### Equity in quality

The training of the health workers is considered as an important way for enhancing health and of helping people access to health care. Our research also paid special attention to the rationality of the distribution of personnel quality. The proportion of health workers with undergraduate degree or above was small in the 190 CHS centers (26.7%), which implied that the skill and technical competency of a certain proportion of health workers might not meet the residents' demands and expectations. Because health policies focus more on the fairness of quantity more than quality, more highly educated doctors prefer to work in economically developed areas, which results in further inequity in quality. Our result showed that there was a greater level of inequitable distribution of health workers with undergraduate degree or above than total health workers. This implied that areas providing better work conditions and higher income also attracted a disproportionately large share of high quality health workers, while disadvantaged areas might have both lower densities of workers and less-educated workforces. To achieve the ‘real fairness’, the central government should keep an eye on the “quality fairness” rather than the numbers.

### Strengths and limitations

Our study has two strengths. Firstly, we used detailed survey data of each CHS centers to provide updated and accurate date instead of national census data which was subject to measurement errors and incompleteness such as misreporting and undercounting. Besides, previous studies mainly evaluated the rural-urban difference in distribution of health care resources instead of the differences within the urban areas. Our study filled the gap in this field.

Two limitations must be mentioned. The main limitation of our study is that it is a sampling research rather than national census. But, our sample was randomly drawn from different regions, which might well represent the inequity status of health workforces in China. Follow-up studies can be conducted in other provinces to confirm the consistency of results. Moreover, our study was a cross-sectional research in 2011, Further researches should include the comparison of the distribution over time to evaluate the impact of government policy.

## Conclusions

The study focused on quantity and quality equity on health workers distribution in urban CHS. Although China has made huge improvements in coverage of community health services over the last decade, our study showed that there were still significant disparities in the distribution of health workers across the provinces in China, especially nurses and highly educated workers. It is suggested that the results of this study should be considered in making decisions on the community health service system by policy makers in Mainland China.

## Supporting Information

S1 Data **The original database.** There are all the raw data of this study to calculate the Gini coefficient. The others census data can be found in the web site of National Bureau of Statistics of the People's Republic of China.
(XLS)
